# Comparative Proteomic Analysis of Two Contrasting Maize Hybrids’ Responses to Low Nitrogen Stress at the Twelve Leaf Stage and Function Verification of *ZmTGA* Gene

**DOI:** 10.3390/genes13040670

**Published:** 2022-04-11

**Authors:** Yafei Wang, Nan Wang, Songtao Liu, Anyi Dong, Tinashe Zenda, Xinyue Liu, Jiao Li, Huijun Duan

**Affiliations:** 1 State Key Laboratory of North China Crop Improvement and Regulation, Hebei Agricultural University, Baoding 071001, China; wyf360536991@163.com (Y.W.); wangnan@hebau.edu.cn (N.W.); m15028293845@163.com (S.L.); 18331220513@163.com (A.D.); tzenda@hebau.edu.cn (T.Z.); lxy1696164468@163.com (X.L.); lj337871790219@163.com (J.L.); 2 North China Key Laboratory for Crop Germplasm Resources of the Education Ministry, Hebei Agricultural University, Baoding 071001, China; 3 Department of Crop Genetics and Breeding, College of Agronomy, Hebei Agricultural University, Baoding 071001, China; 4 College of Agriculture and Forestry, Hebei North University, Zhangjiakou 075000, China

**Keywords:** low nitrogen tolerance, DAPs, morphological characteristics, *Zea mays* L.

## Abstract

Nitrogen is one of the essential nutrients for plant growth and development. However, large amounts of nitrogen fertilizer not only increase the production costs, but also lead to serious environmental problems. Therefore, it is particularly important to reduce the application of nitrogen fertilizer and develop maize varieties with low nitrogen tolerance. The aim of this study was to determine the phenotypic and proteomic alterations of maize affected by nitrogen deficiency and to elucidate the molecular and physiological mechanisms underpinning maize tolerance to low nitrogen. Two maize hybrids with contrasting low nitrogen tolerance were used as the experimental materials. Maize plants were grown under different nitrogen application levels (N0 and N240) and proteomic analysis performed to analyze leaf differentially abundant proteins (DAPs) under different nitrogen conditions. The results showed that under the nitrogen deficiency condition, the nitrogen content, leaf dry weight, leaf area, and leaf area index of XY335 decreased by 15.58%, 8.83%, 3.44%, and 3.44%, respectively. However, in the variety HN138, the same parameters decreased by 56.94%, 11.97%, 8.79%, and 8.79%, respectively. Through proteomic analysis, we found that the low nitrogen tolerance variety responded to low nitrogen stress through lignin biosynthesis, ubiquitin-mediated proteolysis, and stress defense proteins. Transmembrane transporters were differentially expressed in both hybrids after low nitrogen treatment, suggesting that this was a common response to low nitrogen stress. Using bioinformatics analysis, we selected the key candidate gene (*ZmTGA*) that was assumed to respond to low nitrogen stress, and its function was characterized by maize mutants. The results showed that when compared with normal nitrogen treatment, the root length of the mutants under low nitrogen treatment increased by 10.1%, while that of the wild-type increased by 14.8%; the root surface area of the wild type under low nitrogen treatment increased by 9.6%, while that of the mutants decreased by 5.2%; the root surface area of the wild type was higher than that of the mutant at both nitrogen levels; and the activities of glutathione and guaiacol peroxidase enzymes in the mutant were lower than those in the wild-type under low nitrogen treatment. In summary, the mutant was less adaptable to a low nitrogen environment than the wild type. Our results provide maize genetic resources and a new direction for a further understanding of maize response to low nitrogen stress.

## 1. Introduction

Maize (*Zea mays* L.) is one of the most important crops in the world [1]. As a typical C4 plant, its yield potential is much higher than that of wheat (*Triticum aestivum* L.) and rice (*Oryza sativa* L.) [2]. Maize is essentially used to produce human food, animal feed, and biofuel. Therefore, it is of great significance to ensure the stable and sustained increase of maize yield to ensure world food security.

Nitrogen is one of the essential macro nutrient elements in maize production. It plays a crucial role as an essential component of biomolecules in plant life including proteins, nucleic acids, and secondary metabolites. Additionally, N is an important component of cell walls, membranes, and other structures [3,4]. Particularly in maize, nitrogen is important at the twelve leaf (V12) stage, where the plant is in a rapid growth phase and is transitioning to the reproductive phase. At this stage, the plants would have formed about 60% of dry matter and the inflorescence has begun to undergo floret differentiation. Therefore, it is a critical period for maize ear number formation and a critical period for nitrogen fertilization [5]. Insufficient nitrogen supply can affect plant growth and yield. Thus, it is imperative that farmers ensure adequate application of nitrogen fertilizer to crop plants at this stage. 

It is well-known that in agricultural production, a large amount of nitrogen fertilizer is applied to increase crop yields. However, with the increase in nitrogen fertilizer, the yield effect begins to decline, and nitrogen use efficiency becomes significantly lower than 50% [6]. Furthermore, a lot of fertilizer not only increases the economic costs, but also leads to increasingly serious environmental problems such as soil hardening and acidification, eutrophication of water bodies, increase in acid rain, reduction in soil microbial diversity, increased precipitation of heavy metals, and air pollution, etc. [7]. In the face of the dual challenges of food insecurity and environmental degradation, it is of great significance to reduce the amount of nitrogen fertilizer and breed new crop varieties with high nitrogen use efficiency as well as high and stable yields [8]. Therefore, increasing crop tolerance to nitrogen deficiency and improving nitrogen use efficiency at the molecular level is critical for crop breeders.

In order to cope with nitrogen deficiency in the external environment, plants have a variety of adaptive response strategies [9]. At present, several studies on the low nitrogen tolerance of maize have mostly focused on morphological indicators, physiological, and biochemical aspects [10]. Studies have shown that compared with low nitrogen sensitive hybrids, the LAI of low nitrogen tolerant maize hybrids under low nitrogen stress was larger, while the leaf chlorophyll content and total nitrogen content decreased less [11]. Ding et al. [12] found that compared with the old varieties, the reasons for the new varieties to maintain larger plant and grain weight under the condition of nitrogen deficiency were the slower decrease in photosynthetic capacity and the maintenance of phosphoenolpyruvate carboxylase activity and chlorophyll content. Some studies have shown that higher activities of nitrate reductase, glutathione sulfur transferase, nitrogen oxidoreductase, glutamate dehydrogenase, and peroxidase can be used to resist low nitrogen stress [13]. Roots are the main organ for nitrogen uptake. The growth and distribution of roots are determined by genetic characteristics and also influenced by environmental factors [14]. Studies have shown that nitrogen-efficient hybrid varieties have higher aboveground and underground biomass, a deeper root distribution, longer root length, and root active absorption area at lower nitrogen application rates [15]. These morphological indices and physiological and biochemical indices can be used as the criteria for evaluating the crop genotypes’ tolerance to low nitrogen.

TGA (TGACG motif-binding factor) transcription factors are a very important group in the bZIP (basic leucine zipper, bZIP) family [16]. They can specifically bind to the activation sequence -1 (as-1) with TGACG as the core and activate or inhibit the transcription of downstream target genes, thus playing important roles in the plant defense response against biotic or abiotic stresses. Studies have shown that TGA family members interact with different regulatory factors such as non-expressor of pathogenesis-related genes 1 (NPR1), glutaredoxins (GRX), ethylene response factor (ERF), and activated transcription factor 2 (ARR2), which are involved in multiple disease-resistance or stress resistance, and signal transduction pathways such as salicylic acid, jasmonic acid, ethylene, and cytokinin [17,18,19,20]. Glutathione-S-transferase (GST) and cytochrome P450 are two kinds of enzymes that are extremely abundant in plants and are widely involved in stress response. The promoters of cytochrome P450 encoding gene *CYP81D11* and several GST genes all contain the activation sequence -1 (as-1) motif. Studies have shown that *TGA2*, *TGA5*, and *TGA6* in Arabidopsis thaliana can interact with these gene promotors [21,22,23]. More importantly, the expressions of nitrate transporter genes *NRT2.1* and *NRT2.2* were significantly decreased in the TGA/TGA4 double mutant and significantly increased in the *TGA1* or *TGA4* overexpression plants. *TGA1* can directly bind to the promoters of NRT2.1 and NRT2.2 [24]. Transgenic Arabidopsis thaliana with overexpression of *AtTGA4* can significantly improve root development under low nitrogen stress, and the expressions of *NRT2.1* and *NRT2.2* and nitrate reductase genes *NIA1* and *NIA2* are significantly increased, thus improving plant nitrogen transport and assimilation [25].

Proteins are the implementers and executors of functions; therefore, proteomics studies will ultimately help to dissect the possible relationship between protein changes and plant stress tolerance. Proteomic methods are becoming a powerful tool for the comprehensive identification of stress and stress response proteins in plants [26]. Tandem mass tag (TMT) is one of the most powerful methods for quantitative analysis of differential proteins with the highest throughput and minimum systematic error. TMT technology allows for simultaneous labeling of 10 samples and the comparison of protein expression between 2–10 groups of samples at the same time, providing a more accurate digital signal, higher detection flux, and a wider detection range. This technique has become a powerful tool for studying plant stress responses [27].

In order to understand the physiological and molecular responses of maize under low nitrogen stress, this study used the TMT technique to detect changes in the proteome of maize tolerant hybrid line Xianyu 335 (XY335) and sensitive hybrid line Huanong 138 (HN138) at the late vegetative (V12) stage. Additionally, the function of the *ZmTGA* gene in response to low nitrogen stress was preliminarily studied using maize mutants. These results will lay a theoretical foundation and practical significance for proteomic analysis of maize low nitrogen stress and candidate genes related to low nitrogen tolerance in the future.

## 2. Materials and Methods

### 2.1. Plant Material and Experimental Design 

Two maize hybrids with significantly different low nitrogen tolerance characteristics were selected as the test materials in the field experiment. Based on previous research, Xianyu 335 (XY335) is a low-nitrogen efficient (LNE) hybrid, and Huanong 138 (HN138) is a low-nitrogen nonefficient (LNN) hybrid. In both seasons, the LNE hybrids had enhanced N acquisition, biomass production, and yield capacity under N-deficient conditions than the other hybrid groups [28]. The field experiment was conducted at Xinji Experimental station (43°31′ N, 124°48′ E), Shijiazhuang City, China. The soil was sampled from the top 20 cm of the top layer. The soil properties were as follows: organic matter 18.21 g·kg^−1^, alkaline hydrolysis nitrogen 85.27 mg·kg^−1^, available phosphorus 44.38 mg·kg^−1^, and available potassium 186.37 mg·kg^−1^. The soil testing methods followed those of Page et al. (Walkley and Black, 1965). Two nitrogen supply levels: N0 (0 kg·ha^−1^) and N240 (240 kg·ha^−1^). N0 and N240 were designated as N-deficient and N-sufficient conditions, respectively. Urea (46% N) was used as the N source. The dosage of phosphorus (P_2_O_5_) and potassium (K_2_O) were 90 kg·ha^−1^ and 120 kg·ha^−1^, respectively. Other management measures were the same as those in the conventional fields. 

### 2.2. Measurement of Physiological Indices

At the V12 growth stage of maize, three representative plants were selected from each field and the leaf area was measured. The length and width of each leaf were measured using a ruler, and the leaf area and LAI were calculated. Three representative plants were selected from each field. The samples were heated at 105 °C for 30 min and then dried at 70 °C to determine their dry weight. After measuring dry weight, they were ground into powder for the N content assay using a modified Kjeldahl digestion method.

### 2.3. Proteomic Analysis of Two Maize Varieties

There were four treatment combinations: XY335 with N-sufficient control (XYC), XY335 with N-deficient treatment (XYT), HN138 with N-sufficient control (HNC), and HN138 with N-deficient treatment (HNT). Each of the treatment combinations had three biological replicates. Therefore, a total of 12 samples were used for proteomic sequencing. At the 12-leaf stage, we collected the uppermost fully expanded leaf samples for proteomic analysis. Sequencing was performed at Shanghai Majorbio Bio-pharm Technology Co., Ltd. The total protein of all samples was extracted, the protein was digested into peptides, and the peptides were labeled with TMT reagent. Then, the samples were mixed together and analyzed by mass spectrometer. Spectra were searched using Protein Discoverer^TM^ Software 2.2 against the maize database (131585s). Peptide spectral matches were validated based on q-values at a 1% false discovery rate (FDR). Proteins can be identified by the sequence of a unique peptide. A unique peptide refers to a peptide sequence that is specific/unique to a protein group and can represent that protein group. Proteins containing at least one unique peptide were used for subsequent analysis. Student’s t-test was used to analyze the differentially abundant proteins (DAPs), the proteins with a fold-changes >1.2 (up) or <0.83 (down) (*p*-value < 0.05) were considered to be statistically significant DAPs. Gene Ontology (GO, http://www.geneontology.org/, accessed on 15 December 2021) analysis was performed for functional annotation and classification of the identified DAPs. The DAPs were assigned to various biological pathways by the Kyoto Encyclopedia of Genes and Genomes (KEGG, http://www.genome.jp/kegg/, accessed on 15 December 2021) databases. Moreover, a hypergeometric test was used to perform GO and KEGG enrichment analysis.

### 2.4. RNA Extraction and Quantitative Real-Time PCR(qRT-PCR)

Total RNA was isolated from non-stressed and stressed leaves of the two hybrids (XY335 and HN138) using the Omini Plant RNA Kit (DNase I) (CWBIO, Beijing, China) based on the manufacturer’s instructions. Total RNA samples were reverse-transcripted to cDNA using the HiFiscript cDNA Synthesis Kit (CWBIO, Beijing, China). We randomly selected fifteen DAPs and designed gene-specific primers for the quantitative real-time polymerase chain reaction assay using Primer Premier 5 Designer software. Real-time PCR was performed on Light Cycler^®^ 96 using 2X M5 HiPer SYBR Premix EsTaq (Mei5bio, Beijing, China). A steady and constitutively expressed maize gene *GAPDH* (accession no. X07156) was used as the internal reference gene to normalize gene expression data, together with the forward primer (GAPDH-F: 5′-ACTGTGGATGTCTCGGTTGTTG-3′) and reverse primer (GAPDH-R: 5′-CCTCGGAAGCAGCCTTAATAGC-3′). Each sample had three technical replicates. The relative expression levels were calculated with the 2^−ΔΔCT^ method [29].

### 2.5. Bioinformatics Analysis of ZmTGA

The 2000 bp sequence upstream of the *ZmTGA* gene was used as the promoter of the *ZmTGA* gene in maize. The *ZmTGA* promoter sequence was downloaded from the Zea mays refgen_V4-maize from the phytozome database (https://phytozome-next.jgi.doe.gov/, accessed on 3 December 2021). The PLACE database (http://bioinformatics.psb.ugent.be/webtools/plantcare/html/, accessed on 3 December 2021) was used to predict the cis-acting elements. The theoretical molecular weight and isoelectric point were predicted using the website ExPASy (https://web.expasy.org/protparam/, accessed on 6 December 2021). Cell-PLoc 2.0 (http://www.csbio.sjtu.edu.cn/bioinf/Cell-PLoc-2/, accessed on 6 December 2021) was used to predict protein subcellular localization.

### 2.6. Function Verification of ZmTGA Gene

Hydroponic experiments were carried out in the greenhouse to verify the function of the *ZmTGA* gene. Wild-type maize B73 inbred (named TGA) and *ZmTGA* gene mutant seeds (named tga) were used as experimental materials. Maize mutants were ordered from a website called the Maize EMS induced Mutant Database (MEMD) (http://www.elabcaas.cn/memd/index.php, accessed on 1 December 2020) [30]. Maize seeds were disinfected by soaking them in 10% H_2_O_2_ solution for 20 min. The seeds were washed with distilled water and then soaked in distilled water for 12 h. Then, the seeds were wrapped in moist gauze and left in the dark. Once the roots were about 1 cm long, the plants were transferred to quartz sand. When the seedlings reached the two-leaf stage, neat seedlings were selected, the endosperm was removed, and then transferred to a container containing a standardized Hoagland solution. When the seedlings reached the three-leaf stage, different levels of nitrogen were applied into the container. Two nitrogen treatments were used (CK, 4 mM; LN, 0.04 mM). Ca(NO3)_2_ 4H_2_O was used as a nitrogen source. The basic nutrients in hydroponic solutions have been described by Du et al. [31]. In the low N solution, CaCl_2_ was added to maintain the same concentration of calcium with normal conditions. The day temperature of the greenhouse was 26 °C, and the night temperature was 18 °C. The sunshine duration was 16 h. The fresh solution was changed after every two days, where upon the location of the container was also changed. An electric air pump was used to provide ventilation every day.

When the seedlings grew in the nutrient solution for 14 days, the SPAD values were measured with a SPAD-502 portable chlorophyll meter. The SPAD value is positively correlated with leaf chlorophyll content and could be used to predict plant nitrogen status. The SPAD values of at least 10 leaves from the treatment and control groups were measured, respectively. Six seedlings were taken and divided into an aboveground part and underground part, and weighed. Shoot fresh weight, root fresh weight, and root–shoot ratio were calculated, respectively. We used the EPSON root scanner to collect root images, and WinRHIZO (Version2012b, Regent Instruments, Montreal, Canada) software to analyze the images and obtain total root length, average root diameter, and root surface area (SurfArea). The activities of glutathione S-transferase (GST) and peroxidase (POD) were measured using the POD Kit and GST Kit, respectively (Suzhou Comin Biotechnology Co., Ltd., Suzhou, China). 

## 3. Results

### 3.1. Phenotypic and Physiological Characteristics of Two Hybrids under Low-Nitrogen Stress

**Figure 1:**
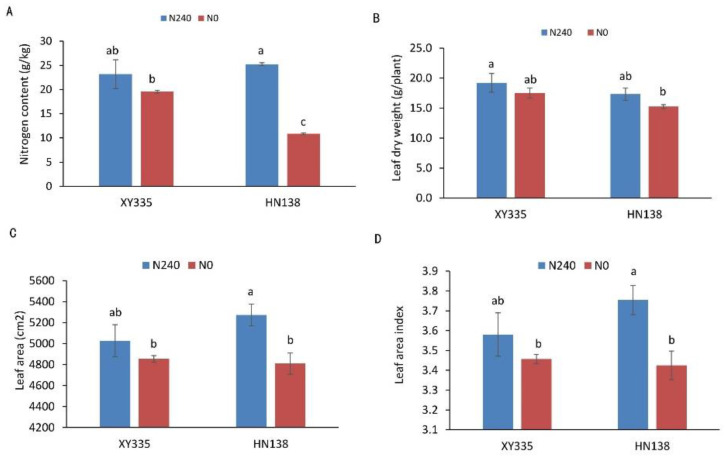
Phenotypic and physiological analysis of two maize hybrids under two nitrogen treatments. (**A**) Leaf nitrogen concentration, (**B**) leaf dry weight, (**C**) leaf area, (**D**) leaf area index. Different letters in the figure indicate significant differences (*p* < 0.05) between hybrids or treatments.

Under low nitrogen stress (N0), leaf area, leaf area index, leaf dry weight, and nitrogen concentration of the two hybrids were all affected to different degrees. Under the nitrogen deficiency condition, the nitrogen content, leaf dry weight, leaf area, and leaf area index of XY335 decreased by 15.58%, 8.83%, 3.44%, and 3.44%, respectively. However, in the variety HN38, the same parameters decreased by 56.94%, 11.97%, 8.79% and 8.79%, respectively. The four indices of low nitrogen tolerance maize hybrid XY335 were not significantly decreased under the N deficiency condition, while the other three indices except for leaf dry weight were significantly decreased in low nitrogen sensitive maize hybrid HN138 (Figure 1). 

### 3.2. Overall View of Maize Leaf Proteins Identified by TMT Analysis

**Figure 2:**
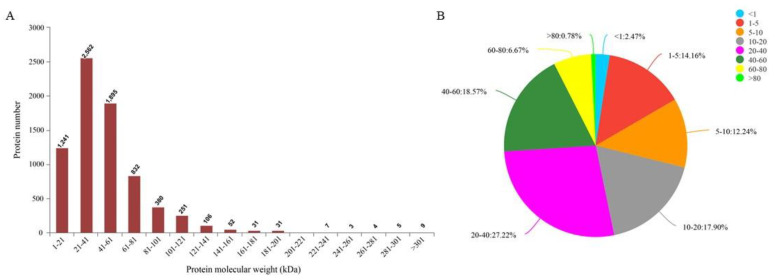
Protein identification and analysis. (**A**) Distribution of protein molecular weight, the abscissa is the distribution range of protein molecular weight, and the ordinate is the number of proteins with corresponding molecular weight. (**B**) Distribution of the protein’s sequence coverage. Each sector represents the proportion of a coverage range. The larger the sector area, the greater the number of proteins with coverage in this range will be. Numbers on the outside of the sector indicate the range of coverage and the proportion of proteins distributed in this region.

Protein sequencing results showed that a total of 909,602 spectra were detected. Through database analysis, a total of 259,102 spectrums were identified, corresponding to 53,547 polypeptides and 7421 proteins. The molecular weight distribution of most proteins ranged from 21 kDa to 61 kDa (Figure 2A). Most protein sequences had a coverage rate of 1–60% (Figure 2B). These results demonstrate the credibility of the protein profile provided by the TMT-labeled mass spectrometer. Proteins containing at least one unique peptide were used for subsequent DAPs analysis. Details of protein identification are shown in Supplementary Table S1.

### 3.3. Analysis of DAPs Observed in Different Experimental Comparisons

Comparative proteomics analysis was used to study the changes in leaf protein profiles of XY335 and HN138 under low nitrogen stress. Paired comparisons before and after treatment (low nitrogen, T, and control, C) were carried out in XY335 (XYT_XYC) and HN138 (HNT_HNC), respectively. In addition, under low nitrogen and normal treatment conditions, a comparative study was carried out between XY335 and HN138, and two comparison groups (HNC_XYC, HNT-XYT) were obtained (Table 1). Under normal nitrogen conditions, a total of 263 DAPs were identified between XY335 and HN138 (XYC_HNC). Under low nitrogen treatment conditions, we found 140 DAPs between XY335 and HN138 (XYT_HNT). In the tolerant variety XY335, 72 proteins showed differential abundance before and after low nitrogen treatment (XYT_XYC); 46 of these DAPs were upregulated. In the low nitrogen sensitive variety HN138, we observed 73 DAPs before and after low nitrogen treatment (HNT_HNC). Of these DAPs, 26 were upregulated and 47 were downregulated. 

**Figure 3:**
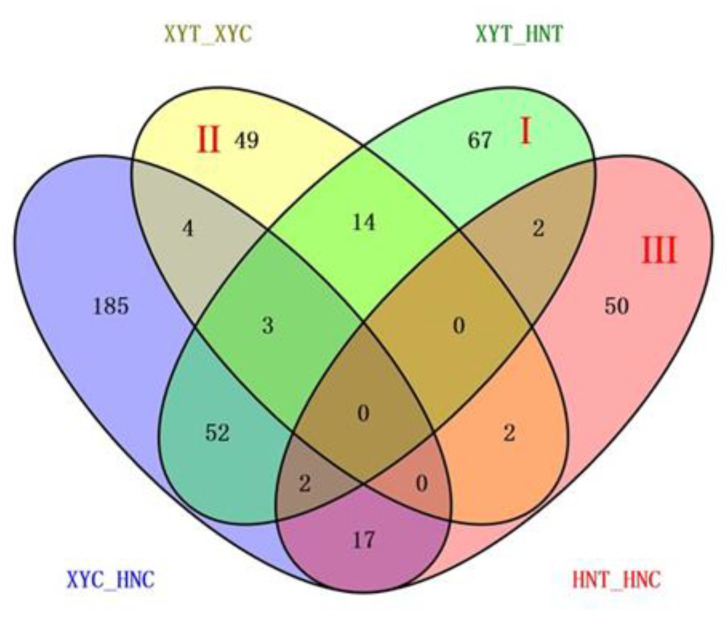
Venn diagram analysis of DAPs identified in the four experimental comparisons. Each compared combination is separated by an underscore (e.g., XYT_XYC, the former is divided by the latter).

With reference to Figure 3, the combination of these four comparisons reflected the influence of different processing conditions on the experiment. The DAPs of the XYT_HNT group were related to both varieties and low nitrogen treatment, which is the group we focused on. After removing the influence of genetic background on gene expression, 67 DAPs in Area I were more associated with low nitrogen treatment. Area II represents the specific DAPs of XYT_XYC, that is, the specific low-nitrogen response DAPs of low-nitrogen tolerant variety XY335. Among the 49 DAPs, 33 were raised and 16 were lowered. There were 50 HNT_HNC specific DAPs in Area III, of which 19 were upregulated and 31 were downregulated. These two regions represent the special coping mechanisms of the two varieties under low nitrogen stress, which may be the reason for the difference in low nitrogen tolerance between the two varieties. Subsequently, we annotated the gene functions of DAPs in these three regions.

### 3.4. Gene Ontology Annotation and Functional Classification

We performed GO annotation to assign GO terms to the DAPs. Furthermore, these GO terms were assigned into biological processes (BP), molecular functions (MF), and cellular component (CC) categories.

**Figure 4:**
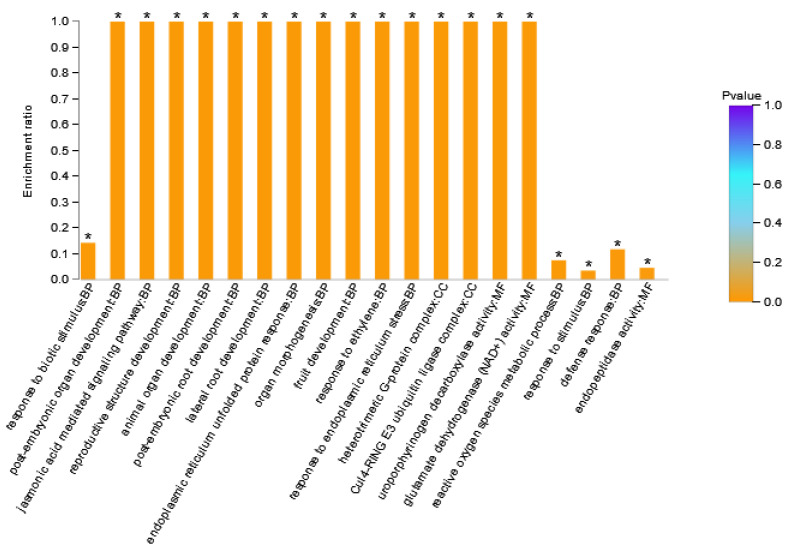
GO enrichment analysis result diagram of Area I. The horizontal axis represents the GO term, and the vertical axis represents enrichment rate, namely the ratio of the protein number enriched in the GO term to the background number annotated to the GO term. The greater the ratio, the greater the degree of enrichment. The column color gradient indicates the significance of enrichment, where *p* < 0.05 was marked as *.

DAPs in Area I were enriched to 66 GO terms (Supplementary Table S2), where BP contained 42 GO terms, MF contained 14 GO terms, and CC contained 10 GO terms. There were 12 stress-related GO terms in the BP category, 11 development-related GO terms, and four signaling pathway related GO terms. The GO terms in the MF category were mainly related to transcription factor activity, peptidase activity, glutamate dehydrogenase, and citrate synthase. GO terms in the CC category were mainly related to the plasma membrane and ubiquitin ligase. The GO terms with a large number of enriched proteins included response to stimulus (six DAPs), response to stress (five DAPs), and endopeptidase activity (four DAPs). Figure 4 showed the 20 GO terms with the highest GO enrichment degree in Area I. The GO term with the highest enrichment significance was response to biotic stimulus. GO terms with the highest enrichment rate were post-embryonic organ development, the jasmonic acid mediated signaling pathway, reproductive structure development, post-embryonic root development, lateral root development, endoplasmic reticulum stressed protein response, organ morphogenesis, fruit development, response to ethylene, response to endoplasmic reticulum stress, heterotrimeric G-protein complex, Cul4-ring E3 ubiquitin ligase complex, and glutamate dehydrogenase (NAD^+^) activity. DAPs with these GO terms might be the key factor of XY335 responding to low nitrogen stress. Among them, Zm00001d033422_P001 (GTP Binding protein 2) participated in 40 GO terms. Zm00001d022542_P003 (Transcription factor TGA6) was the only transcription factor in this grouping, which was assigned to four GO terms.

DAPs in Area II were enriched to 77 GO terms (Supplementary Table S3), where BP contained 37 GO terms, MF contained 25 GO terms, and CC contained 15 GO terms. GO terms assigned to BP were mainly related to the catabolic process. GO terms assigned to MF categories were mainly related to protein binding, DNA binding, protein dimerization activity, transporter activity, and oxidoreductase activity. The GO terms assigned to the CC category were mainly membrane, nucleus, chromosomal part, and DNA packaging complex. Supplementary Figure S1 showed the 20 GO terms with the highest enrichment degree in Area II. The GO terms with the highest enrichment significance were protein heterodimerization activity, DNA packaging complex, protein–DNA complex, chromosomal part, intrinsic component of plasma membrane, and the anchored component of plasma membrane. The GO terms with higher enrichment rate included the valine catabolic process, DNA catabolic process, D-lactate dehydrogenase (cytochrome) activity, glycolate oxidase activity, 3-hydroxyisobutyrate dehydrogenase activity, (S)-2-hydroxy-acid oxidase activity, glycolate dehydrogenase activity, and oxidoreductase activity.

DAPs in Area III were enriched to 57 GO terms (Supplementary Table S4), where BP contained 24 GO terms, MF contained 27 GO terms, and CC contained six GO terms. GO terms in BP were mainly related to single organism process, oxidation–reduction process, transport, response to light intensity, and respiratory electron transport chain. GO terms in MF were mainly oxidoreductase activity, transmembrane transporter activity, and CC mainly included respiratory chain, mitochondrial small ribosomal subunit, and mitochondrial inner membrane. Supplementary Figure S2 showed the 20 GO terms with the highest enrichment degree in Area III. Supplementary Figure S2 showed the 20 GO terms with the highest enrichment degree in Area III. The GO terms with the highest enrichment significance were oxidoreductase activity and respiratory chain. The GO terms with a higher enrichment rate included cellular response to high light intensity, cellular response to light intensity, small ribosomal subunit rRNA binding, mitochondrial small ribosomal subunit, and fumarylacetoacetase activity.

### 3.5. KEGG Pathway Enrichment Analysis of DAPs

In addition to the functional GO annotation, KEGG pathway analysis was also conducted on the significant DAPs, in order to further analyze the functions of the low-nitrogen responsive DAPs. We mapped them to the KEGG database and the DAPs were assigned to various biological pathways. There were five types of KEGG pathways: cellular processes, environmental information processing, genetic information processing, metabolism, and organic systems. 

**Figure 5:**
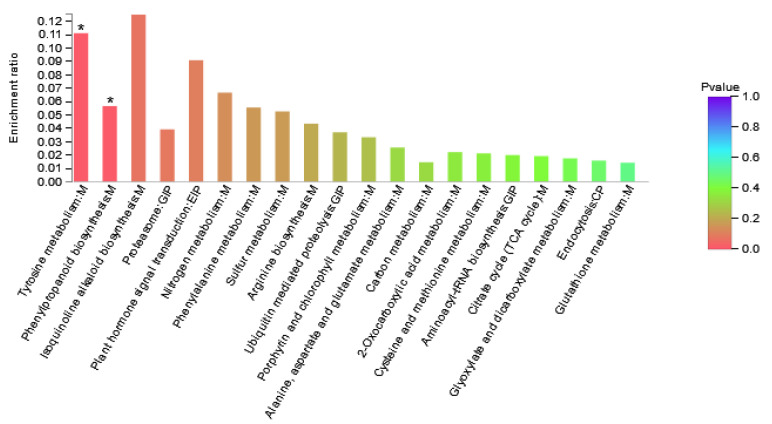
Enriched KEGG pathways of Area I. The abscissa represents the pathway name. The ordinate represents the enrichment rate, which refers to the ratio of the number of proteins enriched in this pathway to the number of proteins annotated into the pathway. The higher the ratio, the greater the degree of enrichment. Column color gradient indicates the significance of enrichment, where *p* < 0.05 is marked with *.

DAPs in Area I were involved in 24 KEGG pathways (Figure 5, Supplementary Table S5). The results showed that the pathways involved in differential proteins mainly included amino acid metabolism, energy metabolism, folding, sorting, and degradation pathways. It is speculated that maize can adjust the adaptability to nitrogen deficiency by changing amino acid metabolism and energy metabolism under the condition of nitrogen deficiency. As shown in the figure, pathways with high enrichment rate included isoquinoline alkaloid biosynthesis, tyrosine metabolism, plant hormone signal transduction, and nitrogen metabolism, and phenylpropanoid biosynthesis. The DAPs involved were Zm00001d022542_P003 (Transcription factor TGA6) and Zm00001d000001_P002 (polyphenol) Oxidase1), Zm00001d022457_P001 (Peroxidase3), Zm00001d046184_P001 (Peroxidase 52), and Zm00001d025984_P001 (glutamic Dehydrogenase2).

DAPs in Area II were involved in 25 KEGG pathways (Supplementary Figure S3, Supplementary Table S6). The KEGG pathways with high enrichment rate included thiamine metabolism, biosynthesis of unsaturated fatty acids, fatty acid biosynthesis, and ubiquitin mediated proteolysis.

DAPs in Area III were involved in 28 KEGG pathways (Supplementary Figure S4, Supplementary Table S7). The KEGG pathways with high enrichment rate included zeatin biosynthesis, circadian rhythm—plant, taurine and hypotaurine metabolism, glycosphingolipid biosynthesis—globo and isoglobo series.

### 3.6. qRT-PCR Analysis

TMT sequencing data were validated by qRT-PCR analysis performed on a selected number of DAPs from different groups. Fifteen gene samples were selected from DAPs of different groups (Supplementary Table S8). The results of the qRT-PCR analysis confirmed our results based on TMT sequencing data. The obtained correlation coefficient (qRT-PCR and TMT-Seq) was 0.848 (Supplementary Figure S5), which proved that the TMT sequencing data we obtained were trustworthy.

### 3.7. Physicochemical Properties and Structure Analysis of ZmTGA

After analyzing the proteomic data, we selected a low nitrogen stress response gene TGA as a candidate gene. This gene was significantly downregulated in the HNT_XYT (Area I) group. After low nitrogen treatment, the expression level in the low nitrogen tolerant variety (XY335) was significantly higher than that in the low nitrogen sensitive variety (HN138). GO enrichment results showed that the gene was enriched in the transcription factor activity (GO: 0003700), regulation of nitrogen compound metabolic process (GO: 0019219), sequence-specific DNA binding (GO: 0043565), and regulation of metabolic process (GO: 0019222). KEGG enrichment analysis found that this gene was enriched on plant hormone signal transduction (pathway ID: zma04075) with a high enrichment rate. Previous studies have found that the bZIP transcription factor *AtTGA4* (TGACG motif-binding factor 4) was induced by both drought and low nitrogen stresses, and that overexpression of *AtTGA4* improved simultaneously in drought resistance and reduced nitrogen starvation in Arabidopsis. Therefore, we chose the *ZmTGA* gene for further research. 

The total length of the *ZmTGA* (Zm00001d022542, GRMZM2G361847) gene was 1818 bp, encoding 333 amino acids and the coding region of 1002 bp residues with a predicted molecular weight of 37.15 kDa and Pi value of 8.90. Subcellular localization prediction showed that the gene was localized in the nucleus. The PLACE database was used to analyze the *ZmTGA* promoter sequence (2000 bp upstream of the gene), and it was found that the promoter contained not only the essential core elements of CAAT-box and TATA-box, but also many elements related to abiotic stress, hormone, and light response (Supplementary Table S9). Abiotic stress related regulatory elements include anaerobic induced ARE, defense, and stress responsiveness cis-acting elements with TC-rich repeats. The hormone-related regulatory elements include CGTCA and TGACG regulated by methyl jasmonate, TCA regulated by salicylic acid, ethylene responsive, gibberellin-responsive TATC box and P-box, ABRE and AAGAA corresponding to abscisic acid, auxin responsive TGA. Several elements involved in light response, AE-box, G-box, I-box, TCT, and MRE, were also found. Sequence analysis of the *ZmTGA* promoter suggested that *ZmTGA* might be a stress-induced and photoperiod regulated promoter.

### 3.8. Phenotypic and Physiological Differences between Mutant tga and Wild-Type TGA in Hydroponic Treatment with Different Concentrations of Nitrate

**Figure 6:**
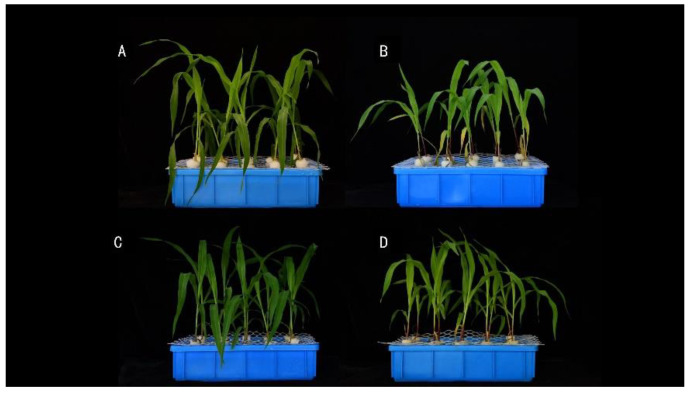
Phenotypic response of maize seedlings under low nitrate (0.04 mM NO^3−^) and optimal nitrate 4 mM NO^3−^) conditions with hydroponics. (**A**) Mutant tga under normal nitrogen treatment conditions. (**B**) Mutant tga under low nitrate. (**C**) Wild-type under normal nitrogen treatment conditions. (**D**) Wild type under low nitrogen treatment.

We verified the function of the *ZmTGA* gene based on the performance of maize tga mutants in the hydroponic condition. In order to adapt to low nitrogen stress, the organisms will spontaneously develop some resistance mechanisms, so their phenotypes will also have many differences. Figure 6 shows the phenotypic results of wild-type TGA and mutant tga at 0.04 mM and 4 mM NO^3−^ for 14 days. It can be seen from the figure that there was no significant difference in the growth status between wild-type and mutant plants under normal nitrogen supply conditions. After low nitrogen treatment, the old leaves of wild-type and mutants turned yellow and senesced prematurely, and the mutant’s leaves turned yellow more than that of the wild-type.

**Figure 7:**
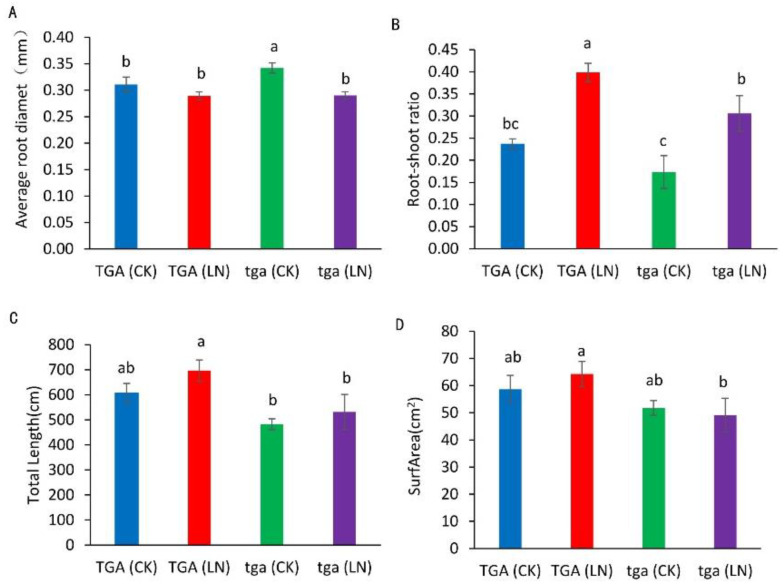
Root differences between wild-type TGA and mutant tga at two nitrogen levels. (**A**) Average root diameter, (**B**) ratio of fresh weight to root shoot, (**C**) total root length, (**D**) root surface area. Different letters indicate significant differences at *p* < 0.05 between different groups according to the Duncan test. CK, 4 mmol L^−1^; LN, 0.04 mmol L^−1^ NO^3−^.

Compared with the normal nitrogen treatment, the root diameter of the wild-type did not decrease significantly under the low nitrogen treatment, while the mutants decreased significantly (Figure 7A). Compared with the normal nitrogen treatment, the root–shoot ratio of the wild-type and mutants increased significantly under the low nitrogen treatment (Figure 7B). Compared with normal nitrogen treatment, the root length of the mutants under low nitrogen treatment increased by 10.1%, while that of the wild-type increased by 14.8%. At both nitrogen levels, the root length of the wild type was higher than that of the mutants (Figure 7C). Compared with the normal nitrogen treatment, the root surface area of the wild type under low nitrogen treatment increased by 9.6%, while that of the mutants decreased by 5.2%. The root surface area of the wild type was higher than that of the mutant at both nitrogen levels (Figure 7D).

**Figure 8:**
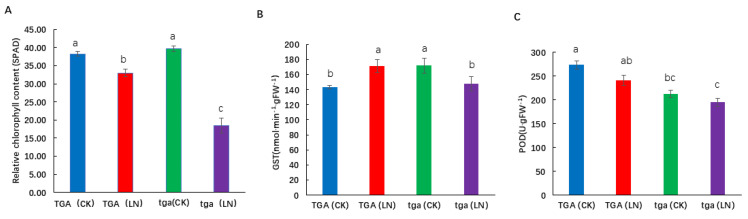
Physiological differences between wild-type TGA and mutant tga at two nitrogen levels. (**A**) Relative chlorophyll content (SPAD), (**B**) Glutathione S-transferase (GST) activity, (**C**) Peroxidase (POD) activity. Different letters indicate significant differences at *p* < 0.05 between different groups according to the Duncan test. CK, 4 mmol L^−1^; LN, 0.04 mmol L^−1^ NO^3−^.

The SPAD value of the wild type and its mutants was significantly reduced at the seedling stage under low nitrogen conditions (Figure 8A). Under low nitrogen conditions, the mutant showed a higher degree of decline than the wild type. Glutathione S-transferase (GST) is a multifunctional protein that plays an important role in cell detoxification and plant growth and development. Under low nitrogen treatment, the activity of GST increased in the wild-type, but decreased in the mutant. The activity of GST in the wild type was significantly higher than that in the mutant (Figure 8B). Higher peroxidase (POD) activity in leaf can reduce the damage caused by membrane lipid peroxidation, delay leaf senescence, and improve the adaptability of leaves to low nitrogen environment. Under low nitrogen treatment, POD activity decreased at both levels, while POD activity of the wild-type was higher than that of the mutant at both nitrogen levels (Figure 8C). In conclusion, the wild-type is more adaptable to low nitrogen stress, and the mutant is more sensitive to nitrogen stress.

## 4. Discussion

### 4.1. Phenotypic Differences in Response to Low Nitrogen Stress between Two Hybrids

Nitrogen is an essential nutrient for plant growth and development [32]. Insufficient nitrogen supply will seriously affect the growth and yield of maize. The results of this study further confirmed that low nitrogen stress inhibited maize plant growth. It can be seen from Figure 1 that under nitrogen stress (N0), leaf area, LAI, leaf dry weight, and nitrogen content of the two hybrids were negatively affected to different degrees. This is consistent with previous research [33]. However, the low nitrogen sensitive hybrid HN138 was more affected by nitrogen stress than the low nitrogen tolerant hybrid XY335. It has been widely reported that photosynthetic capacity is strongly positively correlated with nitrogen content per unit leaf area [34,35,36]. In this study, the unit nitrogen content of low nitrogen tolerance maize hybrid was higher, indicating that the low nitrogen tolerance maize hybrid still had better photosynthetic capacity under nitrogen stress conditions, thus affecting the growth and development of plants and the formation of yield. Nitrogen supply can affect crop growth and yield by controlling LAI and the amount of N per unit leaf area [37]. These indices were less affected by low N stress in low N tolerance maize hybrids. This may be the physiological basis of XY335’s tolerance to low nitrogen.

### 4.2. DAPs Related to ‘Response to Stimulus’ in Two Maize Hybrids after Nitrogen Deficiency Treatment

The GO term containing the largest number of proteins in Area I was response to stimulus. Among the six proteins, two proteins were POD, two were pathogenesis-related proteins, one was the GTP binding protein, and one was the plasma membrane-associated cation-binding protein. POD plays a certain role in scavenging reactive oxygen species in plants, keeping free radicals in cells at a low level and preventing cells from being harmed by free radicals. Studies have shown that high nitrogen use efficiency wheat varieties respond to nitrogen stress by downregulating a stress-related gene annotated as pathogenesis-related protein [38]. GTP binding proteins exist widely in biology and regulate plant development, signal transduction, and biological and abiotic stress responses. Plasma membrane-associated cation-binding protein binds to the plasma membrane and then participates in intracellular signal transduction [39]. This suggests that nitrogen deficiency triggers a general stress response involving many nonspecific responders who are also able to respond to many other abiotic and biological stresses.

### 4.3. DAPs Associated with Low Nitrogen Stress Tolerance

The synthesis pathway of phenylpropane metabolites, especially lignin, plays an important role in plant growth and development and resistance to biological and abiotic stresses [40]. Lignin is one of the main components of the cell wall, which makes plant cells have a hard structure and hydrophobic characteristics, and helps water and minerals to be transported through xylem vascular bundles throughout the plant body. At the same time, lignin is also a synthetic intermediate of plant defense substances. The results of this study showed that phenylpropanoid biosynthesis pathway was significantly enriched in Area I. One DAP, Zm00001d024314_P001 (putative cinnamyl-alcohol dehydrogenase family protein) was upregulated. Cinnamyl alcohol dehydrogenase is an important rate-limiting enzyme in the lignin synthesis pathway of plants. Therefore, after low nitrogen stress treatment, the high lignin metabolism of low nitrogen tolerant varieties can delay cell collapse and play an important role in maintaining the normal cell physiological activities of plants.

Ubiquitin proteasome pathway mediates the degradation of 80–85% of proteins in eukaryotes, and the degradation of non-functional or abnormal proteins can ensure the normal operation of functional proteins [41,42,43]. Currently, many reports have shown that the key enzymes of plant ubiquitination can regulate the adaptability of plants to nutrient stress. Oshrz1 is a ubiquitin ligase that regulates the response and accumulation of iron in plants [44]. NLA (nitrogen limitation adaptation) encodes the E3 ubiquitin ligase, which regulates phosphorus transporters such as PHT1/PHT2 to maintain Pi homeostasis in *Arabidopsis* [45]. Peng et al. [46] showed that mutations in the NLA gene encoding ubiquitin ligase (E3) disrupt Arabidopsis adaptation to nitrogen restriction. In this experiment, ubiquitin mediated proteolysis pathway was enriched in the low nitrogen tolerance variety (Area II), and the key enzymes of ubiquitination was significantly upregulated. Many DAPs in Area I such as proteasome and ubiquitin ligase were also significantly enriched in ubiquitin ligase-related GO term and folding, and sorting and degradation related KEGG. Therefore, ubiquitin mediated proteolysis may be one of the reasons for low nitrogen tolerance maize varieties to adapt to a low nitrogen environment.

The change in membrane system plays a key role in the plant resistance mechanism. The change in membrane structure usually leads to the change of a series of physiological processes such as membrane semi-permeability and enzyme activity bound by the membrane. After low nitrogen treatment, membrane-related proteins were significantly differentially expressed in both hybrids (Area II and III). Plasma membrane intrinsic proteins (PIPs), consisting of six transmembrane domains, play a role in regulating the diffusion of water and small, uncharged solutes. Plasma membrane-associated cation-binding proteins (PCaPs), which lack any transmembrane domain, are involved in intracellular signal transduction through myristoylation binding to the plasma membrane [39]. Both proteins were upregulated in XY335. The V-type ATPase located on the vacuolar membrane plays a role in maintaining the relative stability of cytoplasmic solute and ensuring the normal operation of life activities. After low nitrogen treatment, V-type proton ATPase subunit E3 was downregulated in HN138, while V-Type proton ATPase subunit D was upregulated. In conclusion, nitrogen can regulate intercellular material transport and information transfer by regulating the expression of plant membrane proteins.

### 4.4. Possible Role of ZmTGA Gene in Maize Resistance to Low Nitrogen

In our experiment, TGA transcription factor was significantly differentially ex-pressed. Combined with previous studies, we speculated that this TGA transcription factor might respond to low nitrogen stress, so we conducted further studies on it. Plants overcome the limited nitrogen supply through morphological and physiological adaptations to increase the uptake of nitrogen. Under low nitrogen stress, the chloroplast structure was damaged, the leaves turned yellow, and the content of chlorophyll decreased obviously [47]. In this experiment, low nitrogen stress significantly reduced the leaf SPAD values. Compared with the wild-type, the mutant plants had more yellow leaves and lower SPAD values, and were more affected by low nitrogen stress. Additionally, SPAD values and total nitrogen content decreased more significantly.

When plants grow under low nitrogen conditions, their roots undergo significant changes to adapt to nutrient stress, which is a common finding of plants growing under nutrient stress conditions. Our study showed that at all nitrogen levels, the wild-type had higher root length, root surface area, root–shoot ratio, and root diameter than the mutant and the wild-type had a better ability to absorb nitrogen. Under the condition of low nitrogen stress, the root–shoot ratio increased, indicating that the lower nitrogen supply promoted the growth of maize roots at the seedling stage, and the developed root systems better promoted the growth of the aboveground parts. GST and POD enzymes can reduce the peroxidation damage of cells, maintain the balance between the production and elimination of reactive oxygen species, and improve the resistance of cells [48]. In this experiment, the activities of GST and POD of the wild-type plants were higher than those of the mutant under low nitrogen treatment. Taken together, the wild-type was more adaptable to low nitrogen stress, and the mutant was more sensitive to nitrogen stress, which suggests that *ZmTGA* is a low nitrogen tolerant response gene.

## 5. Conclusions

Based on several physiological indicators, we observed that the relatively tolerant genotype XY335 became less affected by low nitrogen stress conditions than the sensitive hybrid HN138. Through proteomic analysis, we found that the low nitrogen tolerance variety indirectly responded to low nitrogen stress through lignin biosynthesis, ubiquitin-mediated proteolysis, and stress defense proteins. Transmembrane transporters were differentially expressed in both hybrids after low nitrogen treatment, suggesting that this was a common response to low nitrogen stress. Furthermore, we performed function verification of the *ZmTGA* gene through a reverse genetics approach. The results of the hydroponic experiment on wild-type and mutant lines showed that the wild-type was more adaptable to low nitrogen stress, and the mutant was more sensitive to nitrogen stress. In summary, *ZmTGA* is a low nitrogen tolerant response gene.

## Figures and Tables

**Table 1:** Number of DAPs identified in each comparison group.

Comparisons *	Upregulated	Downregulated	Total
HNT_HNC	26	47	73
XYT_XYC	46	26	72
XYT_HNT	80	60	140
XYC_HNC	155	108	263

* Comparisons, differential comparison groups, where the former is divided by the latter; HNT, low nitrogen sensitive variety (HN138) under low nitrogen treatment; HNC, low nitrogen sensitive variety (HN138) under normal nitrogen condition; XYT, low nitrogen tolerant variety (XY335) under low nitrogen treatment; XYC, low nitrogen tolerant variety (XY335) under normal nitrogen conditions.

## Data Availability

The mass spectrometry proteomics data were deposited onto the ProteomeXchange Consortium via the PRIDE partner repository with the dataset identifier PXD028027.

## Supplementary Materials

The following supporting information can be downloaded at: https://www.mdpi.com/article/10.3390/genes13040670/s1, Figure S1: GO enrichment analysis result diagram of Area II; Figure S2: GO enrichment analysis result diagram of Area III; Figure S3: Enriched KEGG pathways of Area II; Figure S4: Enriched KEGG pathways of Area III; Figure S5: Correlation between qRT-PCR and TMT-Seq; Table S1: Details of protein identification; Table S2: Enriched GO terms of Area I; Table S3: Enriched GO terms of Area II; Table S4: Enriched GO terms of Area III; Table S5: Enriched KEGG pathways of Area I; Table S6: Enriched KEGG pathways of Area II; Table S7: Enriched KEGG pathways of Area III; Table S8: Relative expression levels of genes; Table S9: Cis-acting elements contained in the *ZmTGA* promoter sequence.
