# Supplementary material for: Comparative Proteomic Analysis of Two Contrasting Maize Hybrids’ Responses to Low Nitrogen Stress at the Twelve Leaf Stage and Function Verification of ZmTGA Gene
Source: Genes (Basel). 2022 Apr 11;13(4):670. doi: 10.3390/genes13040670 (PMC9030517; doi:10.3390/genes13040670)
Supplement: Supplementary file 1 [file genes-13-00670-s001.zip › genes-1625869-supplementary/supplementary materials/table/Supplementary Table S8.pdf]

Supplementary Table S8 qRT-PCR and TMT-Seq values of 15 genes

ID	qRT-PCR	TMT-Seq
Zm00001d014341(XYT_XYC)	0.38	0.39
Zm00001d015366(XYT_XYC)	0.82	0.30
Zm00001d015366(XYT_HNT)	0.69	0.48
Zm00001d047843(XYT_XYC)	0.18	0.30
Zm00001d047843(XYT_HNT)	0.29	0.37
Zm00001d013706(XYT_XYC)	0.22	0.53
Zm00001d045193(XYT_XYC)	0.77	0.71
Zm00001d045193(HNT_HNC)	0.55	0.41
Zm00001d038163(HNT_HNC)	0.47	0.43
Zm00001d041917(XYT_XYC)	-0.45	-0.42
Zm00001d050965(XYT_XYC)	-0.19	-0.40
Zm00001d006421(HNT_HNC)	-0.52	-0.44
Zm00001d031749(HNT_HNC)	-0.55	-0.36
Zm00001d000035(XYT/XYC)	-0.47	-0.44
Zm00001d014383(XYT/XYC)	-0.82	-0.45
