# Supplementary material for: Comparative Proteomic Analysis of Two Contrasting Maize Hybrids’ Responses to Low Nitrogen Stress at the Twelve Leaf Stage and Function Verification of ZmTGA Gene
Source: Genes (Basel). 2022 Apr 11;13(4):670. doi: 10.3390/genes13040670 (PMC9030517; doi:10.3390/genes13040670)
Supplement: Supplementary file 1 [file genes-13-00670-s001.zip › genes-1625869-supplementary/supplementary materials/table/Supplementary Table S9.pdf]

classification	element	function	number
core elements	TATA-box	core promoter element around -30 of transcription start	20
	CAAT-box	common cis-acting element in promoter and enhancer regions	26
Abiotic stress related regulatory elements	TC-rich repeats	cis-acting element involved in defense and stress responsiveness	3
	ARE	cis-acting regulatory element essential for the anaerobic induction	4
Hormone-related regulatory elements	ABRE、AAGAA-motif	cis-acting element involved in the abscisic acid responsiveness	5
	TGA-element	auxin-responsive element	4
	P-box、TATC-box	cis-acting element involved in gibberellin-responsiveness	4
	CGTCA-motif、TGACG-motif	cis-acting regulatory element involved in the MeJA-responsiveness	4
	TCA-element	cis-acting element involved in salicylic acid responsiveness	3
	ERE	ethylene responsive	2
Light response-related elements	AE-box、G-box、	cis-acting regulatory element involved in light responsiveness	15

	I-box、<br>TCT-motif、<br>MRE		
