# Supplementary figures and images for: Comparative Proteomic Analysis of Two Contrasting Maize Hybrids’ Responses to Low Nitrogen Stress at the Twelve Leaf Stage and Function Verification of ZmTGA Gene
Source: Genes (Basel). 2022 Apr 11;13(4):670. doi: 10.3390/genes13040670 (PMC9030517; doi:10.3390/genes13040670)

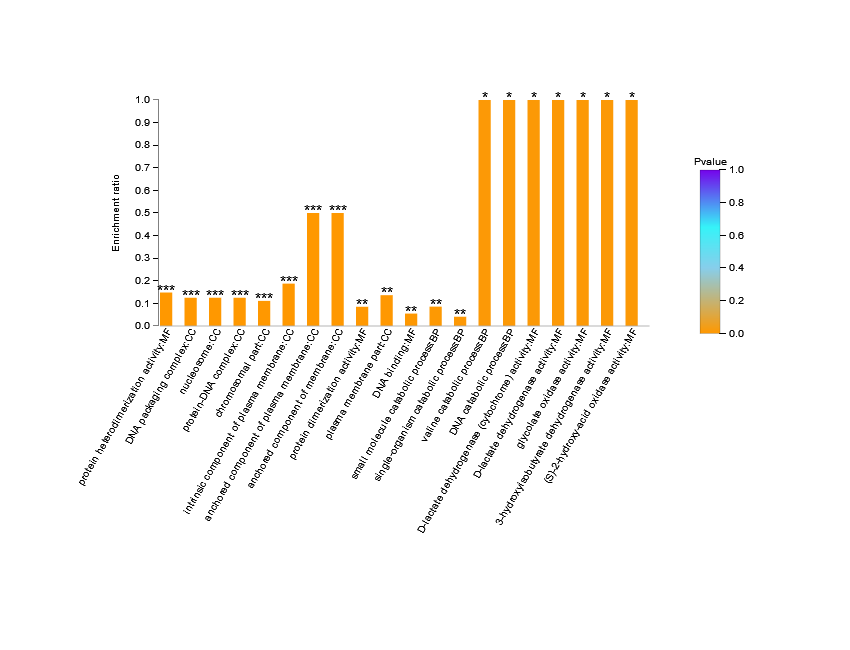

Supplement: Supplementary file 1 [file genes-13-00670-s001.zip › genes-1625869-supplementary/supplementary materials/figure/Supplementary Figure S1.png]

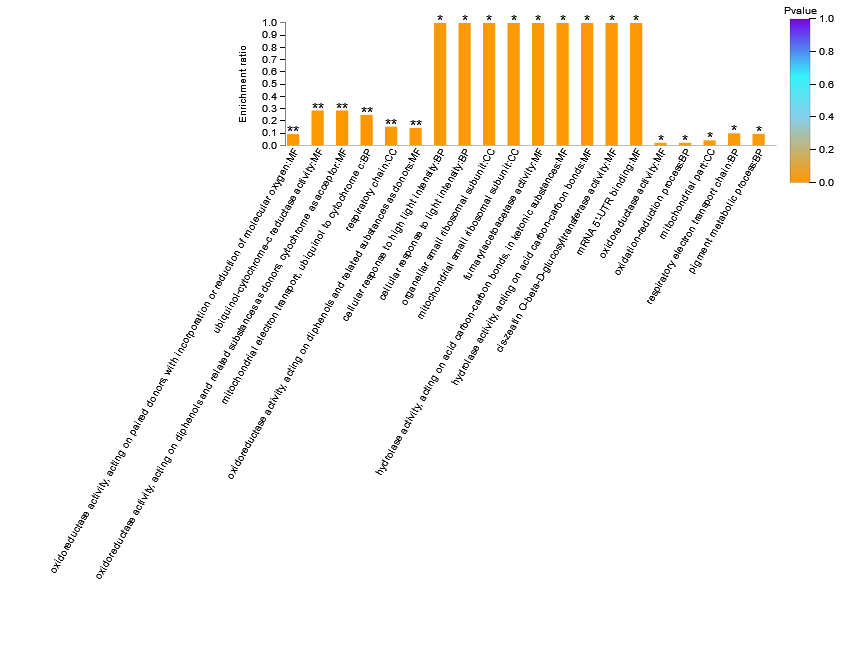

Supplement: Supplementary file 1 [file genes-13-00670-s001.zip › genes-1625869-supplementary/supplementary materials/figure/Supplementary Figure S2.png]

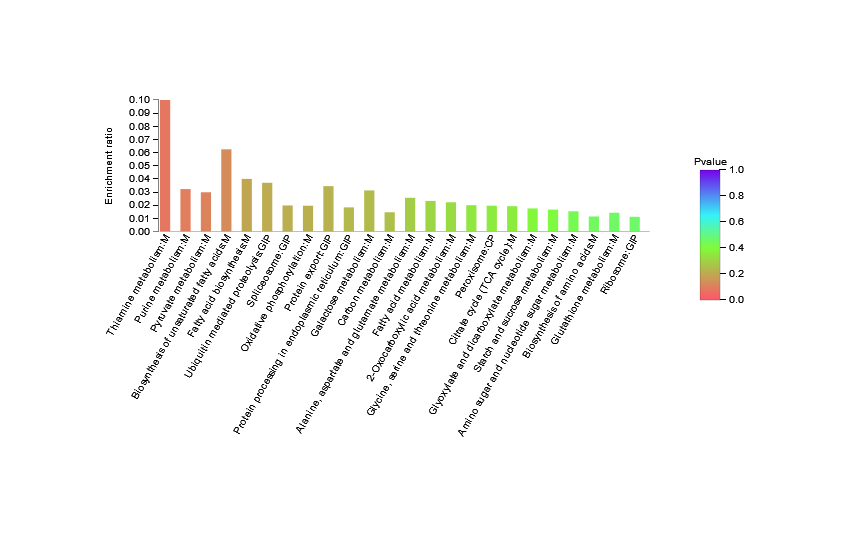

Supplement: Supplementary file 1 [file genes-13-00670-s001.zip › genes-1625869-supplementary/supplementary materials/figure/Supplementary Figure S3.png]

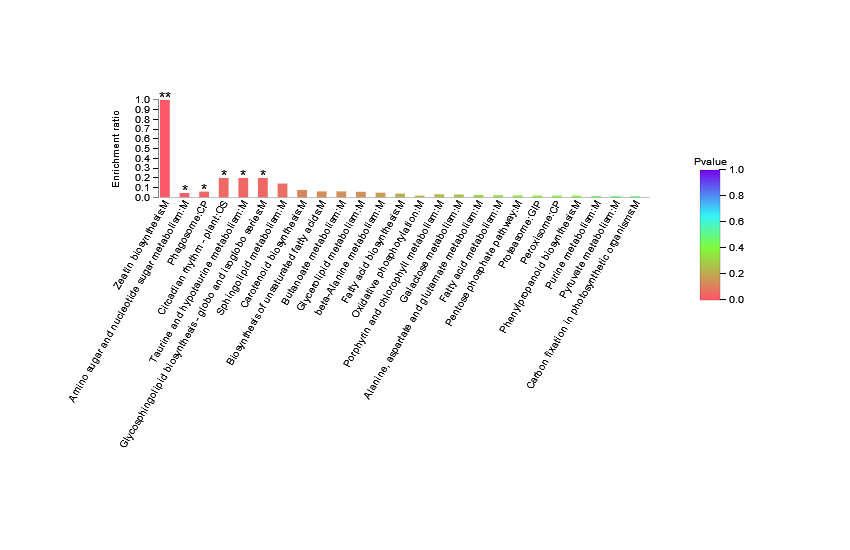

Supplement: Supplementary file 1 [file genes-13-00670-s001.zip › genes-1625869-supplementary/supplementary materials/figure/Supplementary Figure S4.png]

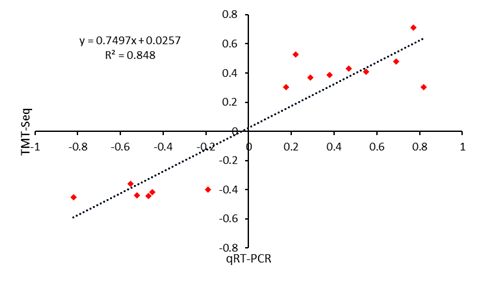

Supplement: Supplementary file 1 [file genes-13-00670-s001.zip › genes-1625869-supplementary/supplementary materials/figure/Supplementary Figure S5.PNG]
